# Scoping literature review and focus groups with healthcare professionals on psychosocial and lifestyle assessments for childhood obesity care

**DOI:** 10.1186/s12913-022-08957-5

**Published:** 2023-02-07

**Authors:** L. W. Koetsier, E. van den Eynde, E. G. A. H. van Mil, M. van der Velde, R. de Vries, C. A. Baan, J. C. Seidell, J. Halberstadt

**Affiliations:** 1 Department of Health Sciences, Faculty of Science, Vrije Universiteit Amsterdam, Amsterdam Public Health Research Institute, Amsterdam, Netherlands; 2 Erasmus MC, University Medical Center Rotterdam, Obesity Center CGG, Rotterdam, Netherlands; 3 Department of Paediatrics, Jeroen Bosch Hospital, PO Box 90153, 5200 ME, s-Hertogenbosch, Netherlands; 4 Maastricht University, Brightlands Campus Greenport Venlo, Maastricht, Netherlands; 5 Public Health Service of Amsterdam, PO Box 2200, 1000 CE, Amsterdam, Netherlands; 6 Medical Library, Vrije Universiteit, Amsterdam, The Netherlands; 7 Tilburg University, Tranzo, Tilburg School of Social and Behavioural Sciences, Tilburg, Netherlands

**Keywords:** Psychosocial and lifestyle diagnostics, Paediatric obesity, Primary health care, Healthcare providers

## Abstract

**Background:**

Childhood obesity is a complex disease resulting from the interaction of multiple factors. The effective management of childhood obesity requires assessing the psychosocial and lifestyle factors that may play a role in the development and maintenance of obesity. This study centers on available scientific literature on psychosocial and lifestyle assessments for childhood obesity, and experiences and views of healthcare professionals with regard to assessing psychosocial and lifestyle factors within Dutch integrated care.

**Methods:**

Two methods were used. First, a scoping review (in PubMed, Embase, PsycInfo, IBSS, Scopus and Web of Science) was performed by systematically searching for scientific literature on psychosocial and lifestyle assessments for childhood obesity. Data were analysed by extracting data in Microsoft Excel. Second, focus group discussions were held with healthcare professionals from a variety of disciplines and domains to explore their experiences and views about assessing psychosocial and lifestyle factors within Dutch integrated care. Data were analysed using template analysis, complemented with open coding in MAXQDA.

**Results:**

The results provide an overview of relevant psychosocial and lifestyle factors that should be assessed and were classified as child, family, parental and lifestyle (e.g. nutrition, physical activity and sleep factors) and structured into psychological and social aspects. Insights into how to assess psychosocial and lifestyle factors were identified as well, including talking about psychosocial factors, lifestyle and weight; the professional-patient relationship; and attitudes of healthcare professionals.

**Conclusions:**

This study provides an overview of psychosocial and lifestyle factors that should be identified within the context of childhood obesity care, as they may contribute to the development and maintenance of obesity. The results highlight the importance of both what is assessed and how it is assessed. The results of this study can be used to develop practical tools for facilitating healthcare professionals in conducting a psychosocial and lifestyle assessment.

**Supplementary Information:**

The online version contains supplementary material available at 10.1186/s12913-022-08957-5.

## Introduction

### Urgency and complexity of childhood obesity

In the Netherlands, in 2020, 12.2% of the children aged 4–17 years had overweight and 2.5% had obesity [1]. Childhood obesity is associated with a range of short-term and long-term physical health problems and psychosocial problems [2, 3]. Moreover, childhood obesity is regarded as a chronic disease that tracks into adulthood [4]. It is a complex disease resulting from the interaction of multiple underlying factors, including personal characteristics (e.g. genetic, hormonal, physical and psychological) and environmental factors (e.g. socio-economic, cultural and physical environments) that can influence lifestyle behaviour and lead to the development or maintenance of childhood obesity [5–7]. Adequate management of childhood obesity calls for taking into account biomedical factors (e.g. genetic factors, comorbidities, extent of overweight or obesity), psychological factors (e.g. self-image, mood, well-being) and social factors (at various levels, including contact with peers, school or authorities) [8, 9].

### Context of the Dutch healthcare system

The importance of conducting an assessment of potentially interacting biomedical, psychosocial and lifestyle factors has been internationally recognised in clinical guidelines [10–13]. The Dutch ‘National model integrated care for childhood overweight and obesity’ describes the assessment of psychosocial and lifestyle factors, which is an essential step as part of the integrated care process. The psychosocial and lifestyle assessment is conducted by a coordinating professional (CP) [14–16]. The CP role can be fulfilled by various professionals from different disciplines and domains (e.g. healthcare domain, social domain). In many cases, it is fulfilled by a professional in the local youth healthcare (YHC) system [15, 16]. The YHC system is based on a municipal or regional infrastructure in which all children 0–19 years of age receive frequent medical check-ups and referrals [17].

### Assessment of psychosocial and lifestyle factors

In a previous study, we examined a supporting assessment tool that CPs can use as a guide for obtaining information about factors that may contribute to the development and maintenance of obesity and obtaining a broad view of children and their family circumstances [18, 19]. In that study, we also identified several potential improvements to be made in the further development of the tool, including the addition of in-depth questions for assessing psychosocial factors, the inclusion of an instructional guide explaining how to use the assessment tool and the adaptation of the tool to make it more age-specific [19]. In addition, the CPs who were interviewed for that study expressed a need for more knowledge about the complexity of obesity and the development of age-appropriate visual materials for conducting psychosocial and lifestyle assessments [19]. Because the study was conducted amongst only fourteen CPs, broader insight is needed with regard to the experiences and views of healthcare professionals (HCPs) from a variety of disciplines and domains with assessing psychosocial and lifestyle factors within the integrated care. Such insights could help to optimise the tool and to assist HCPs in the provision of personalised childhood obesity care.

### Aim of the study

The combination of the designated CP role, the conduction of the psychosocial and lifestyle assessment, mentioned required improvements based on an earlier study and a need from practice for more knowledge and materials makes this study necessary as broader insight is needed [19].

Therefore, the aim of this study is to investigate available literature on psychosocial and lifestyle assessments for childhood obesity and experiences and views of HCPs with regard to assessing psychosocial and lifestyle factors within Dutch integrated care for childhood overweight and obesity.

In order to address the aim, this study centers on two research questions:

1. What national and international scientific literature is available on psychosocial and lifestyle assessments for childhood obesity, and which factors do these assessments address?
2. What are experiences and views of HCPs with regard to assessing psychosocial and lifestyle factors within Dutch integrated care for childhood overweight and obesity?

## Methods

The research process was iterative. To explore the first research question, a scoping review was performed by systematically searching databases for available national and international scientific literature on psychosocial and lifestyle assessments for childhood obesity and describing the factors that are addressed in these studies by doing a deductive analysis. For the second research question, online focus groups were organised with Dutch HCPs from a variety of disciplines and domains in order to explore their experiences and views with regard to assessing psychosocial and lifestyle factors within the Dutch integrated care by doing an inductive analysis.

### Literature

#### Search strategy

A literature search was performed based on the Preferred Reporting Items for Systematic Reviews and Meta-Analyses (PRISMA) statement (www.prisma-statement.org) [20].

To identify all relevant publications, we conducted systematic searches in the bibliographic databases PubMed, Embase.com, APA PsycInfo (EBSCO), IBSS (ProQuest), Scopus and Web of Science from inception to 21 January 2022, in collaboration with a medical information specialist (RdV). The following terms were used (including synonyms and closely related words) as index terms or free-text words:

“Overweight”, “Obesity”, “Children”, “Patient history”, “Anamnesis”, “Psychosocial aspects”, “Lifestyle”.

Duplicate articles were excluded. All languages were accepted. The full search strategies for all databases are provided in the supplementary material.

#### Selection of articles

Studies were included if they met the following criteria: children with obesity aged 0–19 years and a description of an assessment of psychosocial and/or lifestyle factors, or systematic review of nutrition and/or physical activity assessments. We excluded studies if they: (i) were limited to only one dimension (e.g. nutrition, physical activity or medical assessment); (ii) concerned assessments related to patients who were screened for eligibility for bariatric surgery; (iii) were aimed exclusively at assessing motivation; (iv) were editorials, letters, legal cases or interviews. When full-text versions were not available, we contacted authors in an attempt to obtain complete information. Abstracts in languages other than English were translated.

Studies were screened in two stages using the Rayyan systematic review software. First, all relevant titles and abstracts were screened for eligibility (LK) using the inclusion and exclusion criteria, with the first 1000 titles and abstracts independently screened by one of the authors (EvdE). Differences in judgment were resolved through a consensus procedure. The two authors noticed complete agreement, therefore only one author (LK) screened all articles. If relevant, the full-text article was checked for the eligibility criteria. Second, the full-text articles were evaluated independently by two authors (LK and EvdE) for further review. A Microsoft Excel database for data management was created based on an iterative process. LK and EvdE discussed the eligibility, and differences were discussed until consensus was reached.

#### Data synthesis and analyses

For each article, the following data of the full-text articles were extracted in Microsoft Excel by spreadsheet: (i) year of publication; (ii) title; (iii) summary; (iv) target group; (v) study design; (vi) setting in which the assessment was conducted; (vii) how often and by whom the assessment was conducted; (viii) the format of the assessment; (ix) how the assessment was conducted by the HCPs; and (x) the content of the assessment.

The data synthesis was done by LK and EvdE and consisted of searching for consistency of patterns across the extracted data in Microsoft Excel and making comparisons between the extracted data of the studies with similar methodologies. Similarities were observed as a pattern and supplemented with any important distinguishing information. For example, nine articles focused on parent and family factors as part of the assessment which was seen as a similarity, whereas two articles only focused on child factors which was seen as a difference. Differences in how the data was analysed by screening the extracted data were discussed within the research team (LK, EvdE, CB, JS and JH) until consensus was reached.

### Focus groups

#### Participants

Professionals were recruited through the researchers’ professional national network and approached based on varieties in their professional background (healthcare practice, science and policy). The inclusion criterion for participation were knowledge of the supporting assessment tool and, preferably, the Dutch integrated care. Professionals were approached by telephone or by email and asked if they would be willing to participate in the study. Participants received a €10 webshop voucher.

Each participant received an information letter stating the reasons for conducting the research, and all provided recorded informed consent within the Zoom session to participate in this study. The study protocol was approved by the Medical Ethical Committee of the Amsterdam University Medical Center (METC number 2019.511).

#### Data collection

The focus group sessions were held between November 2020 and February 2021. Due to COVID-19 restrictions the sessions were conducted online using Zoom and lasted one and a half hour to 2 h. Each group consisted of four to nine participants. The sessions were conducted by two researchers with prior training in carrying out qualitative research. One of the researchers (LK) moderated the sessions, and another (EvdE) was an observer during the sessions. The focus group topic guide for each session was developed by LK and EvdE in consultation with the research team [see Additional file 1). The focus group topic guides were provided with input on the format of the assessments and the content of the assessment from national and international scientific literature (evidence based) and supplemented with themes from practice (practice based) based on gaps and needs as a result of an earlier performed study [19]. Professionals were asked to provide their availability and the interviews were scheduled based on when most professionals were available. Five focus group sessions were scheduled, with the order of the first four sessions based on the availability of the participants, the final focus group consisted of presenting the interim results and asking for additional input based on the first four focus groups. The focus groups (FG) were intended to generate insight into:

- General experiences and views of CPs with the assessment (FG1)
- Embedding of the assessment within the Dutch integrated care model (FG2)
- Experiences with and views on other assessments (FG3)
- Experiences with and views on the assessment of professionals other than CPs (FG4)
- Embedding of the assessment within the practice of the Dutch integrated care by presenting the interim results and asking for additional input (FG5)

The sessions included interactive methods using the Mentimeter interactive presentation software to generate useful data and to receive input from each participant [available in Dutch on request]. The content of the discussion was audio-recorded and transcribed verbatim. Transcripts were summarised and sent to all participants, who were allowed time to complete or refine their statements as a member check.

#### Data analysis

Template analysis was used to thematically organise and analyse the data of the focus groups (inductive analysis) [21–23]. First, the researchers became familiar with the data by reading the transcripts, summaries, Mentimeter data, additions mentioned in the Zoom chat and field notes. Subsequently, for the initial template, LK performed the preliminary coding of the data, with a subset coded independently by EvdE (open coding). Emerging themes were organised into meaningful clusters (axial coding), and hierarchical relationships were defined (selective coding). LK and EvdE discussed discrepancies until consensus was reached with additional input from the research team (LK, EvdE, CB, JS and JH) and the initial version of the coding template based a subset of the data was defined. The coding template was applied to the remaining data. After necessary modifications and revisions, the template was finalised and applied to the full data set. The data were analysed using MAXQDA 2020 software. The Consolidated Criteria for Reporting Qualitative research (COREQ) were used to guide the reporting of the qualitative findings and has been included as supplementary information (see Additional file 5) [24].

## Results

### Literature

#### Selection and description of included articles

**Fig. 1:**
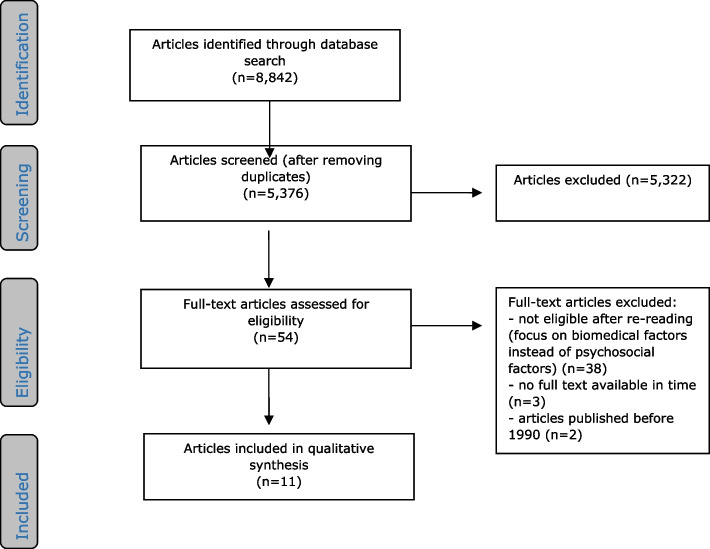
Flowchart for the search and selection procedure for articles

The literature search generated a total of 8842 references: 1,755 in PubMed, 3478 in Embase.com, 532 in PsycInfo, 70 in IBSS, 1755 in Scopus and 1252 in Web of Science [see Additional file 2]. After removing duplicate references, 5376 references remained. The flow chart for the search and selection procedure is presented in Fig. 1.

The 11 included articles are listed in Table 1. Two articles focused only on child factors [27, 30], whereas the others also focused on parent and family factors as part of the assessment [25, 26, 28, 29, 31–35]. Seven articles incorporated the broader healthcare process, including the assessment and the referral and treatment or weight management [26–29, 32–34].

**Table 1:** Description of included articles

Author	Year of publication	Title	Study type	Target population	Targeted professionals	Setting
Barlow et al. [25]	2014	Assessment of the obese child or adolescent	Literature review	Children or adolescent	Primary care providers	Not specified
Bauer et al. [26]	2011	Assessment and management of obesity in childhood and adolescence	Literature review	2–18 years	Primary care providers	Not specified
Chung & Rhie [27]	2021	Severe obesity in children and adolescents: metabolic effect, assessment and treatment	Literature review	Children and adolescents	Medical and health professionals	Paediatric setting
Johansen et al. [28]	2015	Danish clinical guidelines for examination and treatment of overweight and obese children and adolescents in a paediatric setting	Clinical guideline	Children and adolescents	Paediatricians, clinical dieticians, nurses, psychologists, social workers, physiotherapists	Paediatric setting
Jull et al. [29]	2011	Clinical guidelines for weight management in New Zealand adults, children and young people	Clinical guideline	2–18 years	Primary healthcare providers	Primary care setting
Krebs et al. [30]	2007	Assessment of Child and Adolescent Overweight and Obesity	Literature review	Children and adolescents	Physicians and other healthcare professionals/clinicians	Not specified
Phan et al. [31]	2018	Impact of Psychosocial Risk on Outcomes among Families Seeking Treatment for Obesity	Prospective study	4–12 years	Not specified	Weight management clinic
Raatz & Gross [32]	2021	Clinical assessment and treatment of early onset severe obesity	Literature review	0–5 years	Not specified	Clinical setting
Schumann et al. [33]	2002	Preventing paediatric obesity: assessment and management in the primary care setting	Literature review	Children and adolescents	Primary care providers	Primary care
Styne et al. [34]	2017	Paediatric obesity – assessment, treatment, and prevention: endocrine society clinical practice guideline	Clinical guideline	Children and adolescents	Paediatricians	Paediatric setting
Varkula et al. [35]	2009	Assessment of overweight children and adolescents	Book chapter	Children and adolescents	Mental health professionals	Speciality clinic

#### What to include in a psychosocial and lifestyle assessment

Factors that could potentially contribute to the development and maintenance of childhood obesity are presented in Table 2. Assessment factors have been classified as child, family, parental and lifestyle factors and structured into psychological and social aspects. An extensive table with the original description of the factors has been included as supplementary information [see Additional file 3]. Although all articles described biomedical factors (e.g. anthropometric methods) as part of the assessment, these factors were not included in the present study [25–35]. In general, the articles devoted greater attention to biomedical factors than to psychosocial factors.

**Table 2:** Assessment categories, aspects and factors from the included studies

*Author*				Barlow et al	Baur et al.	Chung & Rhie	Johansen et al.	Jull et al.	Krebs et al.	Phan et al.	Raatz & Gross	Schumann et al.	Styne et al.	Varkula et al.
*Year*				*2014*	*2011*	*2021*	*2015*	*2011*	*2007*	*2018*	*2021*	*2002*	*2017*	*2009*
*Category*	*Factors*	*Aspect*	*Subfactor*											
Psychosocial	Child	Psychological	Depression	X	x	x	x	x	x	x		x	x	xx
			Disordered eating	X	x	x	x		x			x	x	x
			Anxiety	X		x	x		x			x	x	
			Self-esteem	X	x		x		x			x		x
			Body image	X			x		x			x		x
			Adverse events					x	xx	x				x
			Weight management		x			x				xx		x
			Consequences of weight					x				x		x
			History of weight		x									x
			Other				x				x		x	x
		Social	Bullying	x	x		x	x					x	x
			Education				x		x			x	x	x
			Social interaction						x	x		x		xxx
			Loneliness				x	x					x	
			Other			x							x	
	Family	Social	Functioning	x	x		x			xx	x	x	x	x
			Culture	x	x		x	x						
			Social support							x	x	x		x
			Family perception							x		x	x	
			Parenting style	x						x		x		
			Relationships		x					x				x
			Rules		x									x
			Other	x										x
	Parent	Psychological	Mental well-being		x					xx			x	x
			Other							x				
		Social	Financial situation	x	x					xx	xx	xx		
Lifestyle	Nutrition	Food intake	Drinks	xx	x	x	x	X	xx				x	
			Fast food		x	x	x	xx	x				x	
			Portion size	x		x	x		x				x	x
			Snacks	x	x	x	x		x					x
			Fruit & vegetables	x			x		x				x	
			General diet								x	x		x
			Breakfast		x				x					
			Appetite			x	x							
		Psychological	Readiness to change	x				x	x			x		x
			Self-efficacy	x					x	x				x
			Other	x								x		x
		Social	Routine	x	x									xx
			Eating outside the home	x					x					x
			Frequency	x					x				x	
			Location of meal	x										x
			Other	x	x						x			xx
	Physical activity	Physical activity	Usual amount of time					x	x		x	x	x	x
			Sports	x	x		x		x			x		
			Unstructured PA	xx	x		x		x					
			Transportation	x	x		x		x					
			Routine PA	x					x			x		
			Other	xxx	x									
		Sedentary behaviour	Access to screen	x	x	x			x			x	x	
			Screen time	x	x		x					x	x	
			Usual amount of time					x	x		x	x		x
			Other		x							x		
		Psychological	Self-efficacy	x					x	x				x
			Readiness to change	x				x	x			x		
			Enjoyment	x										xx
			Other											xx
		Social	Family activities	x	x				x					
			Access	x					x					
			Support	x					x					
			Other						x					
	Sleep	Sleep behaviour	Disorders	x	x	x	x	x	x					
			Disturbances		x		x	xx	x					
			Routine		x		x	x						x
			Amount of sleep			x					x			
		Social	Hygiene					x						

Number of times mentioned in an article: ‘x’: once; ‘xx’: twice; ‘xxx’: three or more times

*Psychosocial assessment*

The extent to which psychosocial factors were described in the articles varied from elaborate descriptions, including assessment techniques and examples of questions for both children and parents [35], to a table containing brief descriptions of psychosocial problems [30].

Child factors

Psychological factors of the child included weight-related depression and anxiety, eating disorders, self-esteem and body image, which are specific to the assessment of childhood obesity, as opposed to more generic assessments [25–30, 33–35]. Additionally, three articles included the identification of adverse events, such as major family events and a history of abuse or neglect [30, 31, 35].

In nine articles, social concerns (e.g. bullying, loneliness or problems with social interaction) were identified as social factors of the child [25, 26, 28–31, 33–35]. Five articles noted to the importance of considering education (e.g. school avoidance and school performance) [28, 30, 33–35].

Family factors

One major aspect of social factors of the family identified in most assessments is the importance of determining family functioning in terms of environment, structure, composition or other aspects [25, 26, 28, 31–35]. Four assessments included ethnicity and cultural factors, albeit to varying extents [25, 26, 28, 29]. For example, one assessment in the form of a clinical guideline was specifically intended for minority populations (i.e. Maori, Pacific and South Asian populations) [29].

Parental factors

Four articles reported psychological factors of the parents that related to mental well-being. These articles differed in the extent to which the factors were described in relation to childhood obesity. Factors reported included adverse events, mental health concerns and eating disorders [26, 31, 34, 35]. The social factor of the parents that was most prominently identified as being important to take into account was financial situation [25, 26, 31–33]. Six articles did not consider social parental factors [27–30, 34, 35].

*Lifestyle assessment*

Ten articles included the assessment of nutrition and physical activity as part of the lifestyle assessment [25–30, 32–35]. The majority of the lifestyle assessments focused on the current lifestyle behaviour, and one article also focused on the adoption of desired healthy lifestyle behaviours by the entire family (nutrition education and physical activity) in addition to current lifestyle behaviour [30].

The specificity and extent of nutritional and physical activity assessment varied, as did the extent of resources provided to professionals. For example, one nutritional assessment offered a structured assessment to ensure the inclusion of relevant information concerning details of eating habits, including intake of sugar-sweetened beverages, milk and juices, fruits and vegetables, snacks and fast food, as well as appetite and portion size [28]. Physical activity assessments included details on time spent in a variety of activities or organised sports, transportation to and from school, time spent in sedentary behaviour and screen time per day.

The focus on the psychological and social aspects of nutrition and physical activity varied, and these aspects were not considered in a literature review and a clinical guideline for the examination and treatment of children and adolescents with obesity [27, 28]. Psychological aspects of nutrition and physical activity were mentioned in six assessments and in relation to readiness to change and the level of confidence in the ability to make changes (self-efficacy) [25, 29–31, 33, 35].

The majority of the assessments mentioned sleep behaviour as potentially contributing to excessive weight gain during childhood [25–30, 32, 35]. Most of the articles did not describe sleep patterns as part of lifestyle factors, but often as part of the biomedical assessment. For example, some assessments included the identification of various sleep-related problems, including disordered sleep, obstructive sleep apnoea syndrome and disruptive snoring [25–30].

#### How to conduct a psychosocial and lifestyle assessment

The included articles focused on what to include in a psychosocial and lifestyle assessment. Eight of these articles also paid attention to how to conduct the assessment of psychosocial and lifestyle factors [25, 26, 29–31, 34, 35]. These findings were divided into the three most prominent themes: (a) talking about psychosocial factors, lifestyle and weight; (b) the professional-patient relationship; and (c) attitudes of healthcare professionals.

*Talking about psychosocial factors, lifestyle and weight*

As noted by Barlow et al. and Varkula et al., it is important to communicate sensitively and introduce the topic carefully (e.g. by asking whether a patient or parent has any concern about the child’s weight) [25, 35], as children and parents might feel ashamed and defensive about obesity [25]. They further advised HCPs to use the terms preferred by parents [25, 35]. According to Jull, HCPs should avoid jargon and explain any health terms clearly, in addition to reflecting on their own communication preferences (e.g. the words and tone used; body language) [29].

Four articles paid explicit attention to behaviour change techniques (e.g. goal setting, stimulus control and self-monitoring) and conversational techniques (e.g. motivational interviewing) that should be applied [26, 30, 31, 34]. According to Johansen et al., open-ended questions and reflective listening techniques could help direct communication towards changes in behaviour [26].

*The professional-patient relationship*

The importance of the professional-patient relationship was explicitly mentioned in articles by Jull et al. and Varkula et al. [29, 35]. According to these two articles, early rapport building and a non-judgmental demeanour are of the utmost importance to the ideal assessment and management of childhood obesity. The authors stressed the vital importance of involving the family and engaging with children and families, building enhancing and collaborative relationships, and showing genuine respect [29, 35].

*Attitudes of healthcare professionals*

As noted in studies by Barlow et al., Baur et al. and Jull et al., ideal assessment and management calls for HCPs to adopt an emphatic, supportive, non-judgmental and collaborative attitude [25, 26, 29].

### Focus groups

#### Study characteristics

An overview of the self-reported general characteristics of the focus-group participants is presented as supplementary information [see Additional file 4]. In all, 28 professionals participated in the study, one of who participated in two focus groups. Four other professionals cancelled their participation due to personal circumstances. The mean age of the participants was approximately 45 years, and 25 (89%) of the participants were female.

The participants represented a total of 35 functions, as several participants combined multiple functions. The focus groups included professionals working 15 different positions at a variety of levels in the healthcare system, ranging from community care to secondary care: integrated (or general) care advisors (*n* = 8; 27.6%), YHC nurses (*n* = 6; 17.1%), CPs (*n* = 5; 14.3%), YHC doctor (*n* = 3; 8.6%), paediatricians (*n* = 2; 5,7%), project leaders of the local integrated care (*n* = 2; 5.7%), managers of the local integrated care (*n* = 2; 5.7%), specialised YHC nurses (*n* = 2; 5.7%), professor of nutrition and health (*n* = 1; 2.9%), social worker (*n* = 1; 2.9%), dietician (*n* = 1; 2.9%), researcher (*n* = 1; 2.9%) and trainer and developer of national education for CPs (*n* = 1; 2.9%). An overview of the distribution of positions is provided as supplementary information [see Additional file 4].

The participants represented a total of 29 different organisations, most within the municipal health services (*n* = 12; 41.4%). Other organisations included a municipality (*n* = 3; 10.3%), ‘Youth on a Healthier Weight’ (JOGG) (*n* = 3; 10.3%), a hospital (*n* = 2; 6.9%), the Netherlands Youth Institute (*n* = 2; 6.9%), the Dutch Centre for Youth Healthcare (*n* = 2; 6.9%), a dietician practice (*n* = 1; 3.5%), a university (*n* = 1; 3.5%), a primary school (*n* = 1; 3.5%) and a professional association (*n* = 1; 3.5%). One participant (3.5%) was self-employed. An overview of the organisations represented is provided as supplementary information [see Additional file 4].

#### What to include in a psychosocial and lifestyle assessment

Relevant factors that should be taken into account as part of the assessment have been classified as child, family, parental and lifestyle factors and structured into psychological and social aspects. According to the HCPs, there is no need to assess all these factors in detail at once, the CP makes the decision what is assessed and when it is assessed depending on the situation of the child and family.

*Psychosocial assessment*

Child factors

Factors that participants identified as important to consider focused largely on the well-being of the child, stress and relaxation. The participants also emphasised the importance of discussing the strengths and capabilities of the child and family. Depending on the child’s age (predominantly with children aged 12 years and older), factors such as peer pressure, gaming behaviour and gaming in combination with sleeping were regarded as relevant.

When children go to secondary school, they often have money and go along with the group. Those who can’t afford to buy snacks between meals are in an awkward position, because the rest of the group is going, and they like these things as well. (Focus group 2, R5).

Family factors

Participants emphasised the need to consider various aspects of family functioning, including the family situation and composition (e.g. separated parents and blended families) and mutual relationships between family members. They also mentioned the importance of assessing parenting skills, including the following topics: parental trust, setting clear boundaries, parental attitudes and beliefs with regard to upbringing, parental agreements on parenthood, and the experiences of parents with their own upbringing. According to the participants, co-caregivers (e.g. grandparents, daycare workers) who play a role in childcare and who bear some responsibility for upbringing should also be taken into account, given the critical importance of agreements on upbringing and lifestyle behaviour between caregivers.

Grandparents play an important role in families, and their views often conflict with those of the parents. Many parents are glad when grandparents are willing to take on a caregiving role. When grandparents want to reward children with fast food, sweets or salty snacks that the parents don’t approve of, however, this places the children under pressure. It’s obviously important for these things to be clear. The issue thus often goes beyond children and their direct caregivers to include the environment as well. (Focus group 4, R4, Pos. 53).

Finally, the participants noted that it is helpful to consider the environment of the family (e.g. social support, the networks of the parents, perception of weight by peers and culture).

And there should also be a cultural connection: the meaning of food in a family, sociability, hospitality, et cetera. (Focus group 2, R5, Pos. 96).

Parental factors

Participants noted the importance of considering whether parents have a job and what their work situation is. They also considered it important to talk about the financial possibilities, stress and relaxation of the parents.

I would like to see more attention to stress and relaxation, and what they need in that regard. This refers to factors that children experience as stressful, as well as those that parents see as stressful, as they are not necessarily the same. This is an important distinction. (Focus group 4, R4, Pos. 77).

*Lifestyle assessment*

Given that the focus groups centred on psychosocial factors, and given that participants felt that the current psychosocial and lifestyle assessment places sufficient emphasis on lifestyle factors, no additional lifestyle factors were mentioned.

It can be tempting to focus more on lifestyle issues and less on the underlying psychosocial issues or factors. I think people need more help in order to consider the issue more broadly. (Focus group 1, R4, Pos. 31)

#### How to conduct a psychosocial and lifestyle assessment

The experiences and views of HCPs with regard to the assessment of psychosocial and lifestyle factors also addressed the issue of how to conduct the assessment of psychosocial and lifestyle factors. The findings emerging from the analysis of the focus-group discussions were classified according to the three most prominent themes: (a) talking about psychosocial factors, lifestyle and weight; (b) the professional-patient relationship; and (c) attitudes of healthcare professionals.

*Talking about psychosocial factors, lifestyle and weight*

Participants emphasised the importance of talking about psychosocial factors, lifestyle and weight in order to gain insight into the factors that may contribute to the development and maintenance of obesity and to ensure a contextualised and comprehensive understanding of children with obesity and their circumstances. They specifically highlighted the sensitivity of the topic of obesity and some psychosocial factors. According to the participants, children and their parents may feel guilt and shame because of their weight, possibly leading them to avoid talking about obesity, psychosocial factors, lifestyle and weight, in addition to avoiding future appointments.

*The way you introduce the conversation is important, given the vulnerability associated with obesity. The words used and questions asked are very important, as it can quickly seem like an interrogation. (Focus group 3, R6, Pos. 170).*

*The way you introduce the conversation is important, given the vulnerability associated with obesity. The words used and questions asked are very important, as it can quickly seem like an interrogation. (Focus group 2, R5, Pos. 159).*

In order to prepare children and parents, participants stressed the crucial importance of explaining the need to assess the broader circumstances of children and their families, as they are likely to expect the assessment to focus only on weight and lifestyle. The participants also highlighted the need for CPs to acknowledge and explain the complexity of obesity. More specifically, children and parents should be aware of factors that influence their behaviour and weight. According to the participants, practical tools may help professionals to present interacting factors in a visual, non-judgmental manner, thereby facilitating conversations about psychosocial factors, lifestyle and weight.

*Even if you already know the families, it’s important to explain why you want to take a broader look at the family at that particular moment. (Focus group 5, R8, Pos. 65).*

*We all know that conversations on this topic are difficult. It’s extremely important to explain why we’re asking these questions and what they actually have to do with each other. This is obvious to us, but not necessarily to parents. (Focus group 3, R6, Pos. 114).*

Sufficient knowledge about the complexity of obesity, healthy food and the tools that are available were mentioned as important means of enabling professionals to conduct psychosocial and lifestyle assessments. Communication skills (e.g. applying various conversational techniques, such as motivational interviewing and solution-focused counselling) were also identified as a key element.

*It requires interviewing skills to make contact in an interested, professional manner without going straight for the target. (Focus group 1, R2, Pos. 166).*

Participants felt that it is more difficult to discuss psychosocial factors, lifestyle and weight with families with different cultural backgrounds who either have low literacy or face language barriers, and that the assessment thus needs more attention.

*For parents with language problems, it is sometimes not until the second or third session that they start to understand what I meant when I asked if a dietician had already visited them or if they have had any previous help. I would actually have liked for that to have been the case at the first session. (Focus group 2, R5, Pos. 62) I’ve noticed that assessments are quite difficult when dealing with other cultures. It requires a lot of explanation, especially for people with a different background who don’t speak Dutch. This obviously makes the conversation quite different. (Focus group 2, R3, Pos. 22).*

*The professional-patient relationship*

The participants regarded the professional-patient relationship as important to both the assessment and the management of childhood obesity. Given that it often takes considerable time to build rapport and a trusting relationship with children and their parents, the participants noted that multiple consultations may be required in order to conduct psychosocial and lifestyle assessments. I’ve also noticed that questions can be too daunting for a first conversation. There might still be some resistance if I were to try to address that right away. It sometimes takes several sessions before it’s safe enough. (Focus group 2, R4, Pos. 80).

With regard to the professional-parent relationship, CPs should 1) introduce their role and take time to explain the value of talking about psychosocial and lifestyle factors for both the child and the parents; 2) get to know the family better by asking about and trying to understand their living circumstances; and 3) create clear expectations about the care process.

I also think it’s good for coordinating professionals to introduce themselves: who I am and what I can do for them. This is not always clear to parents. (Focus group 5, R7, Pos. 70).

It’s really helpful to create a bond of trust and to help children and their parents to feel that you’re genuinely interested in them, and not just in the excess weight or how the child is eating and exercising. This completely changes the conversations. (Focus group 4, R4, Pos. 59).

*Attitudes of healthcare professionals*

Participants expressed that conducting a psychosocial and lifestyle assessment requires an attitude shift for most CPs. They stressed the need for CPs to be ‘demand-oriented’ and patient with regard to the priorities and requests of children and their families. Adopting an interested, curious and empathic attitude was considered helpful during the assessment. The participants highly endorsed the use of open-ended questions and engaging in active listening with a non-patronising attitude.

You have to have a particular mindset. You’re asking something completely different of professionals. Everyone might say, ‘Yeah, we know’. They might hear it, and it might sound good, but translating it into action really does ask something of them. (Focus group 1, R6 Pos. 39).

## Discussion

This article is based on scientific literature on childhood obesity assessments and information obtained from focus groups on the experiences and views of healthcare professionals (HCPs) with regard to assessing psychosocial and lifestyle factors that influence the development and maintenance of obesity. The results provide an overview of relevant psychosocial and lifestyle factors classified into four categories—child, family, parents and lifestyle—and structured into psychological and social aspects. The article also highlights the importance of paying attention to sensitivity when talking about psychosocial factors, lifestyle and weight within the context of such assessments, as well as the importance of a good professional-patient relationship and an emphatic, supportive, non-judgmental and collaborative attitude on the part of the HCP.

The scientific literature included in the scoping review reflected differences in the extent of detail in which various assessments address psychosocial and lifestyle factors, as well as with regard to other aspects that are addressed, including the management of obesity and how HCPs should conduct the assessment. Moreover, some of the literature provided an overview of principles of childhood obesity management, in addition to the psychosocial and lifestyle assessment [26, 28, 29]. One explanation for such differences could be that the articles included in the review were targeted at audiences in different healthcare disciplines and settings (e.g. medical professionals and mental healthcare professionals). Furthermore, the information obtained through the assessment might change throughout the healthcare process. In order to identify changes in the circumstances of children and their families, as well as to evaluate intervention outcomes and reconsider treatment goals, it would seem useful to integrate the assessment into the care process and assess psychosocial and lifestyle factors at several different points, rather than using such assessment exclusively as a diagnostic tool [36]. An appropriate assessment aimed at understanding factors that might contribute to the development and maintenance of obesity is essential to improving the efficacy of obesity management [37]. Such assessments can help to identify and address various facilitators and barriers. Studies have indicated that this can enhance the success of obesity treatments, in addition to increasing compliance with and adherence to treatment [38].

As indicated by the results of both the scoping review and focus groups, it is important to facilitate the work of HCPs by explaining how to conduct a psychosocial and lifestyle assessment. This finding is remarkable, given that the study was intended to search for available scientific literature on psychosocial and lifestyle assessments for childhood obesity, and experiences and views of healthcare professionals with regard to assessing psychosocial and lifestyle factors within Dutch integrated care, rather than to generate insight into how such an assessment should be conducted. One possible explanation could be that the notion of addressing psychosocial factors was unfamiliar to HCPs, as well as to children and their families, as standard obesity care to date has not devoted sufficient attention to such factors [39, 40]. The participants in this study felt that conducting a psychosocial and lifestyle assessment is difficult. This might be due to the sensitive nature of the child’s weight and the risk that raising the issue of weight might alienate families or lead them to drop out of treatment [41, 42]. In addition, the results of the scoping review and extensive research has documented the pervasive presence of implicit and explicit bias and stigma relating to weight, including amongst HCPs, and this has an impact on the care that they provide [43–48]. It could also lead children and their parents to avoid or delay healthcare services [45, 49]. Given that bias and stigma relating to weight are driven by insufficient acknowledgement of the complex aetiology of obesity, efforts to build awareness and understanding concerning the complexity of obesity could help to reduce the prevalence of bias against obesity [47]. Visual materials that explain the complexity of obesity could be helpful in this regard, for instance an illustrated tool to support conversation [50]. The results of the scoping review and focus groups further highlight the importance of sensitive communication and a respectful and trustful patient-professional relationship when conducting psychosocial and lifestyle assessments, as these aspects could decrease the likelihood of stigmatisation, thereby making support and care more accessible to children and their parents [51].

The extent to which HCPs feel that they are able to conduct a psychosocial and lifestyle assessment seems to be related to years of work experience, affinity with the issues relating to overweight and obesity, level of knowledge, confidence in one’s own professional skills and the ability to adjust one’s attitude to correspond to individual children and their parents [19]. The role of these elements is well documented in the literature of the scoping review and other literature with regard to discussing a child’s weight, as well as when referring children to treatment and obesity counselling [19, 46, 52]. This is supported by the results of the scoping review and focus groups.

### Limitation and strengths

A limitation of the scoping review is that it did not assess the methodological quality of the included studies as this did not add to the aims of the study. This stud intended to receive as many input and inspiration as possible with regard to psychosocial and lifestyle assessments which can be seen as a strength. In addition, a limitation of the focus groups is that the participants were not randomly selected as the inclusion criteria for participation were knowledge of the supporting assessment tool and, preferably, the Dutch integrated care. Since many of the participants had prior experience within the integrated care, they may not have been representative of all HCPs. Given the possibility of selection bias, the topics of this study might have been perceived differently by professionals with less experience with integrated care (e.g. because they need more support or guidance). However, the qualitative data reflect a variety of HCPs in terms of disciplines and professional experience. Moreover, the use of the Mentimeter interactive presentation software during the focus groups ensured the collection of a wide variety of input, as it allowed for obtaining additional information from each individual participant. Finally, data saturation was achieved, as indicated by the confirmation of the themes and conclusions in the final focus group.

One strength of this study is that two methods were used involving the use of qualitative data from focus groups to expand on and add depth to the results of a systematic literature review. In addition, the use of databases for the fields of healthcare, social work and psychology ensured that the scoping literature review reflects a wide range of literature, thereby enhancing the generalisability of the findings.

### Implications for practice and future research

Although the role of the coordinating professional (CP) is specific to the context of the healthcare system in the Netherlands, the findings may also be relevant to childhood obesity care in other Western countries. Given that the results reflect lessons learned with regard to what should be included in psychosocial and lifestyle assessments and how such assessments should be conducted, they might also be applicable to for adults and for other chronic diseases.

The results of the scientific literature and experiences and views of HCPs are synthesized and used to develop practical tools for HCPs within the context of integrated care in the Netherlands [50, 53, 54]. Developing practical tools for HCPs based on the results of the scoping review and focus groups of this study that corresponds to the national and local contexts within which they provide obesity care might also be relevant for other integrated care contexts, including: (1) examples of questions concerning psychosocial and lifestyle factors; (2) inspiration for obtaining deeper insight into psychosocial and lifestyle factors (e.g. health related quality of life) [55]; (3) suggestions concerning how to talk about psychosocial and lifestyle factors in a positive and structured manner; and (4) guidance for what HCPs should do after the assessment. The involvement of HCPs as well as children and parents in the development of appropriate tools could increase the likelihood of successful implementation. Another practical implication is that age-appropriate visual materials that provide insight into the complexity of obesity should be developed in order to support the process of conducting psychosocial and lifestyle assessments. Finally, it is important for HCPs to be trained to conduct psychosocial and lifestyle assessments, as this could enhance their confidence and skills, while contributing to de-stigmatisation. HCPs will need time to experiment with the tool, and they will need to gain experience with how to personalize to the needs and priorities of individual children and their parents. The practical implications of this study are already being applied within the context of integrated care in the Netherlands [50, 53, 54, 56, 57].

Future research should focus on evaluating psychosocial and lifestyle assessments with HCPs, as well as with children and their parents, in order to identify their needs and wishes, as current research does not adequately reflect their voices. Studies should also address the impact of conducting a psychosocial and lifestyle assessment as part of the integrated care process on the outcomes of care.

## Conclusion

This study provides an overview of psychosocial and lifestyle factors that should be identified and how they should be addressed in order to personalise childhood obesity care as part of integrated care for childhood overweight and obesity. These insights can be translated into practical tools for facilitating HCPs in the process of conducting psychosocial and lifestyle assessments in a sensitive and adequate manner. Future research should evaluate the needs, wishes and experiences not only from the perspective of HCPs, but also from the perspective of children and their families. This will promote continuous learning and thereby the further development of the integrated care and the tools associated with it. In addition, the impact of such developments on the outcomes of care should be monitored.

## Supplementary Information

**Additional file 1. **Focus group sessions topic guides.

**Additional file 2. **Literature search results.

**Additional file 3. **Extensive table containing the original descriptions of factors that may contribute to the development and maintenance of childhood obesity and that should therefore be identified.

**Additional file 4. **Tables.

**Additional file 5. **Consolidated criteria for reporting qualitative studies (COREQ): 32-item checklist.

## Data Availability

All data generated or analysed during this study are included in this published article and the supplementary information files. The data analysed during the current study are available upon reasonable request from the corresponding author (l.koetsier@vu.nl).

## Abbreviations

CP: Coordinating professional
YHC: Youth healthcare
HCP: Healthcare professional
