# Supplementary material for: Scoping literature review and focus groups with healthcare professionals on psychosocial and lifestyle assessments for childhood obesity care
Source: BMC Health Serv Res. 2023 Feb 7;23:125. doi: 10.1186/s12913-022-08957-5 (PMC9903277; doi:10.1186/s12913-022-08957-5)
Supplement: Supplementary file 1 — Additional file 1. Focus group sessions topic guides. [file 12913_2022_8957_MOESM1_ESM.docx]

## Supplementary files

### Additional File 1. Focus group sessions topic guides

#### Focus group session 1, dinsdag 1 december 2020, 9.00 – 11.00 uur

Doel focusgroep 1: brede anamnese voor kinderen met obesitas beter laten aansluiten bij de visie en andere stappen van het landelijk model

Deelvraag 1: Op welke manier kan de brede anamnese voor kinderen met obesitas nog beter aansluiten bij de brede blik/integrale visie van het landelijk model?

Deelvraag 2: Op welke manier kan de brede anamnese voor kinderen met obesitas nog beter aansluiten bij de stappen na stap 2 van het landelijk model?

Bespreekpunten:

- Introductie onderzoek en werkwijze
- Uitgangspunt en doel van de focusgroep: hoe kan de (psychosociale en leefstijl) brede anamnese voor kinderen met obesitas verbeterd worden?
- Openingsvraag
- Kort voorstellen (naam, rol, organisatie, leeftijd) en wat zijn volgens jou de huidige knelpunten mbt de brede anamnese?
- Hoofdvraag
  - Op welke manier kan de brede anamnese voor kinderen met obesitas nog beter aansluiten bij de brede blik/integrale visie van het landelijk model?

Pauze

- - Op welke manier kan de brede anamnese voor kinderen met obesitas nog beter aansluiten bij de stappen na stap 2 van het landelijk model?
- Korte brainstorm suggesties naam tool
- Overige opmerkingen/ aanvullingen deelnemers vanuit genoemde knelpunten openingsvraag
- Afrondende vraag:
- Toevoegingen/belangrijke dingen om mee te nemen die nog niet zijn besproken?
- Zie parkeerpunten
- Vervolg en afsluiten

#### Focus group session 2, dinsdag 3 november 2020, 9.00 – 10.30 uur

Doel focusgroep 2:

Inhoudelijke verdiepingsslag brede anamnese voor kinderen met obesitas.

Hoofdvraag:
Welke vragen moeten worden verdiept of toegevoegd aan de brede anamnese voor kinderen met obesitas vanuit praktijkervaring van de eindgebruiker?

Bespreekpunten:

- Introductie onderzoek en werkwijze
- Uitgangspunt en doel van de focusgroep: hoe kan de (psychosociale en leefstijl) brede anamnese oor kinderen met obesitas verbeterd worden?
- Openingsvraag
- Kort voorstellen en wanneer is deze focusgroep voor jou geslaagd?
- Hoofdvraag

Onderstaande thema’s zijn genoemd in eerder onderzoek naar de ervaringen en behoeftes van centrale zorgverleners in het afnemen van de brede anamnese:  
(1) Meer vragen rondom psychosociale factoren
(2) Leeftijdspecifiekere vragen (kindertaal/ onderscheid ouder-kind)  
(3) Aanvullende vragen 12+ (zelfbeeld, groepsdruk ..)  
(4) Volgorde van de thema’s in de anamneselijst  
(5) Doel/visie (het waarom) achter de vragen in de anamneselijst

- Herkennen jullie je hier in?
- Zijn er nog aanvullingen?
- Prioriteren van de genoemde verbeterpunten
- Herkennen jullie je hier in?
- Welke thema’s missen jullie?
  - Clusteren van de aangevulde thema’s
  - Wat is de ideale situatie?
  - Wat is er nodig om deze ideale situatie te bereiken?
- Overige opmerkingen/ aanvullingen deelnemers vanuit openingsvraag
- Afrondende vraag:
- Gespreksleider toetst de belangrijkste ideeën die naar voren zijn gekomen tijdens de discussie. Zijn er toevoegingen?
- Vervolg en afsluiten

#### Focus group session 3, woensdag 18 november, 9.30 – 11.00 uur

Doel focusgroep: Leren van bestaande brede methodieken in de praktijk

Hoofdvraag: Wat heb je nodig voor een brede anamnese voor kinderen met obesitas, waarbij geput kan worden uit de praktijkervaring/kennis die professionals hebben met andere bestaande brede methodieken.

Bespreekpunten:

- Introductie onderzoek en werkwijze
- Uitgangspunt en doel van de focusgroep: hoe kan de (psychosociale en leefstijl) brede anamnese voor kinderen met obesitas verbeterd worden?
- Openingsvraag
- Kort voorstellen (naam, rol, leeftijd, gemeente, methodiek)
  - - Wat typeert de methodiek waarmee je werkt?
- Hoofdvraag
  - Als we het hebben over de inhoud van de methodiek (bv thema’s, gespreksonderwerpen)
    - Welke thema’s zijn bruikbaar vanuit de methodiek waar jij mee werkt?
      - Via mentimeter (wordcloud), en op basis hiervan de discussie op gang brengen
  - Als we het hebben over de manier van afnemen (bv vragenlijst/thema’s/kaartjes, alleen met kind/samen met ouder, leeftijd kind, gewenste locatie)
    - Wat werkt minder goed (barrières)?
      - Via mentimeter, en op basis hiervan de discussie op gang brengen
    - Wat werkt goed (facilitators)?
      - Via mentimeter, en op basis hiervan de discussie op gang brengen
- Afrondende vraag:
- Gespreksleider toetst de belangrijkste ideeën die naar voren zijn gekomen tijdens de discussie. Zijn er toevoegingen?
- Vervolg en afsluiten

#### Focus group session 4, dinsdag 8 december 2020, 10.00 – 11.30 uur

Doel focusgroep: consulatie ketenprofessionals voor optimaliseren brede anamnese voor kinderen met obesitas

Deelvraag 1: Welke informatie haal jij nu uit de brede anamnese?

Deelvraag 2: Welke informatie zou je willen ophalen uit de brede anamnese?

Bespreekpunten:

Introductie onderzoek en werkwijze

- Uitgangspunt en doel van de focusgroep: hoe kan de (psychosociale en leefstijl) brede anamnese voor kinderen met obesitas verbeterd worden?

Openingsvraag

- Kort voorstellen (naam, rol, organisatie, leeftijd) en op wat voor manier heb jij te maken met de brede anamnese?

Hoofdvragen

- Welke informatie haal jij nu uit een brede anamnese?

Pauze

- Welke informatie zou je willen ophalen uit een brede anamnese?
- Overige opmerkingen/ aanvullingen deelnemers vanuit genoemde knelpunten openingsvraag

Afrondende vraag:

- Toevoegingen/belangrijke dingen om mee te nemen die nog niet zijn besproken?

Vervolg en afsluiten

Focus group session 5, dinsdag 16 februari 2021, 14.30 – 16.00 uur

Doel focusgroep: Toetsen van ‘vertrekpunten’ doorontwikkeling brede anamnese-tool voor kinderen met obesitas.
Deelvraag 1: Kloppen de vertrekpunten en zijn er aanvullingen?

Deelvraag 2: Hoe faciliteren we flexibel gebruik van de tool (bv volgorde van vragen stellen, niet alles in 1 keer, gebruik naar eigen professionele inzicht)

Bespreekpunten:

Introductie onderzoek en werkwijze

- Uitgangspunt en doel van de focusgroep: vertrekpunten doorontwikkeling (psychosociale en leefstijl) brede anamnese voor kinderen met obesitas

Openingsvraag

- Kort voorstellen (naam, rol, organisatie, leeftijd) en hoe heb je te maken met de brede anamnese-tool, wat mag volgens jou niet ontbreken aan de brede anamnese tool?

Hoofdvragen

- Korte toelichting 3 vertrekpunten (bijlage, introductie, inhoud), klopt dit en zijn er aanvullingen?
- Wat hebben professionals nodig om de tool flexibel te gebruiken?
  - Hoe zorgen we ervoor dat het niet lijkt alsof alles in 1 keer uitgevraagd moet worden? Hoe uitputtend gaan we zijn?
  - Belangrijk dat lijst niet op 1 moment gebruikt wordt; hoe en waar in het proces op meerdere momenten benutten?
- Overige opmerkingen/ aanvullingen deelnemers

Afrondende vraag:

- Toevoegingen/belangrijke dingen om mee te nemen die nog niet zijn besproken?

Vervolg en afsluiten
