# Supplementary material for: Scoping literature review and focus groups with healthcare professionals on psychosocial and lifestyle assessments for childhood obesity care
Source: BMC Health Serv Res. 2023 Feb 7;23:125. doi: 10.1186/s12913-022-08957-5 (PMC9903277; doi:10.1186/s12913-022-08957-5)
Supplement: Supplementary file 2 — Additional file 2. Literature search results. [file 12913_2022_8957_MOESM2_ESM.docx]

### Additional File 2. Literature search results

PubMed Session Results (21 Jan 2022)

| Search | Query | Items found |
| --- | --- | --- |
| #7 | #4 AND #5 AND #6 | 1,755 |
| #6 | **"Life Style"[Mesh] OR "Health Behavior"[Mesh] OR "Parenting"[Mesh] OR "life style*"[tiab] OR lifestyle*[tiab] OR behavior*[tiab] OR behaviour*[tiab] OR psychosocial*[tiab] OR psycho-social*[tiab] OR psychological[tiab] OR parenting[tiab]** | 2,105,416 |
| #5 | **"Medical History Taking"[Mesh] OR "Risk assessment"[Mesh] OR anamnes*[tiab] OR "medical history"[tiab] OR "case history"[tiab] OR "intake interview*"[tiab] OR "structured interview*"[tiab] OR "validated interview*"[tiab] OR assessment*[ti] OR screening*[ti] OR assessment*[ot] OR screening*[ot]** | 938,451 |
| #4 | **#1 OR (#2 AND #3)** | 104,868 |
| #3 | **"Overweight"[Mesh] OR obese*[tiab] OR obesity[tiab] OR overweight*[tiab]** | 411,852 |
| #2 | **child*[tw] OR schoolchild*[tw] OR infan*[tw] OR adolescen*[tw] OR pediatri*[tw] OR paediatr*[tw] OR boy[tw] OR boys[tw] OR boyhood[tw] OR girl[tw] OR girls[tw] OR girlhood[tw] OR youth[tw] OR youths[tw] OR toddler*[tw] OR teen[tw] OR teens[tw] OR teenager*[tw] OR puberty[tw] OR preschool*[tw]** | 4,413,846 |
| **#1** | **"Pediatric Obesity"[Mesh]** | 11,582 |

Embase.com Session Results (21 Jan 2022)

| Search | Query | Items found |
| --- | --- | --- |
| #8 | #7 NOT ('conference abstract'/it OR 'conference review'/it) | 3,478 |
| #7 | #4 AND #5 AND #6 | 4,206 |
| #6 | **'lifestyle'/exp OR 'lifestyle modification'/exp OR 'health behavior'/exp OR 'parental behavior'/exp OR 'child parent relation'/exp OR 'life style*':ab,ti,kw OR lifestyle*:ab,ti,kw OR behavior*:ab,ti,kw OR behaviour*:ab,ti,kw OR psychosocial*:ab,ti,kw OR 'psycho-social*':ab,ti,kw OR psychological:ab,ti,kw OR parenting:ab,ti,kw** | 2,583,746 |
| #5 | **'anamnesis'/exp OR 'risk assessment'/exp OR anamnes*:ab,ti,kw OR 'medical history':ab,ti,kw OR 'case history':ab,ti,kw OR 'intake interview*':ab,ti,kw OR 'structured interview*':ab,ti,kw OR 'validated interview*':ab,ti,kw OR assessment*:ti OR screening*:ti OR assessment*:kw OR screening*:kw** | 1,725,623 |
| #4 | **#1 OR (#2 AND #3)** | 139,691 |
| #3 | **'obesity'/exp OR obese*:ab,ti,kw OR obesity:ab,ti,kw OR overweight*:ab,ti,kw** | 706,200 |
| #2 | **child*:ab,ti,kw,de OR schoolchild*:ab,ti,kw,de OR infan*:ab,ti,kw,de OR adolescen*:ab,ti,kw,de OR pediatri*:ab,ti,kw,de OR paediatr*:ab,ti,kw,de OR boy:ab,ti,kw,de OR boys:ab,ti,kw,de OR boyhood:ab,ti,kw,de OR girl:ab,ti,kw,de OR girls:ab,ti,kw,de OR girlhood:ab,ti,kw,de OR youth:ab,ti,kw,de OR youths:ab,ti,kw,de OR toddler*:ab,ti,kw,de OR teen:ab,ti,kw,de OR teens:ab,ti,kw,de OR teenager*:ab,ti,kw,de OR puberty:ab,ti,kw,de OR preschool*:ab,ti,kw,de** | 4,642,878 |
| **#1** | **'childhood obesity'/exp** | 18,979 |

APA PsycInfo (Ebsco) Session Results (21 Jan 2022)

| Search | Query | Items found |
| --- | --- | --- |
| S6 | S3 AND S4 AND S5 | 532 |
| S5 | **DE "Lifestyle" OR DE "Lifestyle Changes" OR DE "Health Behavior" OR DE "Parenting" OR (TI ("life style*" OR lifestyle* OR behavior* OR behaviour* OR psychosocial* OR "psycho-social*" OR psychological OR parenting) OR AB ("life style*" OR lifestyle* OR behavior* OR behaviour* OR psychosocial* OR "psycho-social*" OR psychological OR parenting) OR KW ("life style*" OR lifestyle* OR behavior* OR behaviour* OR psychosocial* OR "psycho-social*" OR psychological OR parenting))** | 1,454,859 |
| S4 | **DE "Patient History" OR DE "Risk Assessment" OR (TI (anamnes* OR "medical history" OR "case history" OR "intake interview*" OR "structured interview*" OR "validated interview*" OR assessment* OR screening*) OR AB (anamnes* OR "medical history" OR "case history" OR "intake interview*" OR "structured interview*" OR "validated interview*") OR KW (anamnes* OR "medical history" OR "case history" OR "intake interview*" OR "structured interview*" OR "validated interview*" OR assessment* OR screening*))** | 243,197 |
| S3 | **S1 AND S2** | 17,230 |
| S2 | **TI (child* OR schoolchild* OR infan* OR adolescen* OR pediatri* OR paediatr* OR boy OR boys OR boyhood OR girl OR girls OR girlhood OR youth OR youths OR toddler* OR teen OR teens OR teenager* OR puberty OR preschool*) OR AB (child* OR schoolchild* OR infan* OR adolescen* OR pediatri* OR paediatr* OR boy OR boys OR boyhood OR girl OR girls OR girlhood OR youth OR youths OR toddler* OR teen OR teens OR teenager* OR puberty OR preschool*) OR KW (child* OR schoolchild* OR infan* OR adolescen* OR pediatri* OR paediatr* OR boy OR boys OR boyhood OR girl OR girls OR girlhood OR youth OR youths OR toddler* OR teen OR teens OR teenager* OR puberty OR preschool*)** | 1,041,502 |
| **S1** | **DE "Overweight" OR DE "Obesity" OR DE "Obesity (Attitudes Toward)" OR (TI (obese* OR obesity OR overweight*) OR AB (obese* OR obesity OR overweight*) OR KW (obese* OR obesity OR overweight*))** | **50,911** |

International Bibliography of the Social Sciences - IBSS (ProQuest) Session Results (21 Jan 2022)

| Search | Query | Items found |
| --- | --- | --- |
| S6 | S3 AND S4 AND S5 | 70 |
| S5 | **MAINSUBJECT.EXACT("Lifestyles") OR MAINSUBJECT.EXACT("Health behavior") OR MAINSUBJECT.EXACT("Parents & parenting") OR ti,ab,su("life style*" OR lifestyle* OR behavior* OR behaviour* OR psychosocial* OR "psycho-social*" OR psychological OR parenting)** | 474,932 |
| S4 | **MAINSUBJECT.EXACT("Patient assessment") OR MAINSUBJECT.EXACT("Risk assessment") OR ti,su(anamnes* OR "medical history" OR "case history" OR "intake interview*" OR "structured interview*" OR "validated interview*" OR assessment* OR screening*) OR ab(anamnes* OR "medical history" OR "case history" OR "intake interview*" OR "structured interview*" OR "validated interview*")** | 61,661 |
| S3 | **S1 AND S2** | 2,456 |
| S2 | **ti,ab,su(child* OR schoolchild* OR infan* OR adolescen* OR pediatri* OR paediatr* OR boy OR boys OR boyhood OR girl OR girls OR girlhood OR youth OR youths OR toddler* OR teen OR teens OR teenager* OR puberty OR preschool*)** | 273,457 |
| **S1** | **MAINSUBJECT.EXACT("Obesity") OR ti,ab,su(obese* OR obesity OR overweight*)** | 7,354 |

Scopus Session Results (21 Jan 2022)

| Search | Query | Items found |
| --- | --- | --- |
| #6 | #3 AND #4 AND #5 | 1,755 |
| #5 | **TITLE-ABS-KEY ("life style*" OR lifestyle* OR behavior* OR behaviour* OR psychosocial* OR "psycho-social*" OR psychological OR parenting)** | 6,698,229 |
| #4 | **(TITLE (anamnes* OR "medical history" OR "case history" OR "intake interview*" OR "structured interview*" OR "validated interview*" OR assessment* OR screening*)) OR (ABS (anamnes* OR "medical history" OR "case history" OR "intake interview*" OR "structured interview*" OR "validated interview*")) OR (AUTHKEY (anamnes* OR "medical history" OR "case history" OR "intake interview*" OR "structured interview*" OR "validated interview*" OR assessment* OR screening*))** | 1,421,868 |
| #3 | **#1 AND #2** | 134,975 |
| #2 | **TITLE-ABS-KEY (child* OR schoolchild* OR infan* OR adolescen* OR pediatri* OR paediatr* OR boy OR boys OR boyhood OR girl OR girls OR girlhood OR youth OR youths OR toddler* OR teen OR teens OR teenager* OR puberty OR preschool*)** | 5,562,594 |
| **#1** | **TITLE-ABS-KEY (obese* OR obesity OR overweight*)** | 562,594 |

Web of Science (Core Collection) Session Results (21 Jan 2022)

| Search | Query | Items found |
| --- | --- | --- |
| #6 | #3 AND #4 AND #5 | 1,252 |
| #5 | **TS=("life style*" OR "lifestyle*" OR "behavior*" OR "behaviour*" OR "psychosocial*" OR "psycho-social*" OR "psychological" OR "parenting")** | 4,541,315 |
| #4 | **TS=("anamnes*" OR "medical history" OR "case history" OR "intake interview*" OR "structured interview*" OR "validated interview*") OR TI=("assessment*" OR "screening*") OR AK=("assessment*" OR "screening*")** | 1,153,994 |
| #3 | **#1 AND #2** | 105,169 |
| #2 | **TS=("child*" OR "schoolchild*" OR "infan*" OR "adolescen*" OR "pediatri*" OR "paediatr*" OR "boy" OR "boys" OR "boyhood" OR "girl" OR "girls" OR "girlhood" OR "youth" OR "youths" OR "toddler*" OR "teen" OR "teens" OR "teenager*" OR "puberty" OR "preschool*")** | 3,074,487 |
| **#1** | **TS=("obese*" OR "obesity" OR "overweight*")** | 500,378 |
