# Supplementary material for: Scoping literature review and focus groups with healthcare professionals on psychosocial and lifestyle assessments for childhood obesity care
Source: BMC Health Serv Res. 2023 Feb 7;23:125. doi: 10.1186/s12913-022-08957-5 (PMC9903277; doi:10.1186/s12913-022-08957-5)
Supplement: Supplementary file 4 — Additional file 4. Tables. [file 12913_2022_8957_MOESM4_ESM.docx]

### Additional File 4. Tables

#### General characteristics of the participants in the focus groups

| N | Focus group | Age (years) | Function | Gender | Organisation |
| --- | --- | --- | --- | --- | --- |
| 1 | 1 | 33 | Project leader of the local integrated care approach, YHC nurse and CP^a^ | Female | Municipal health service |
| 2 | 1 | 40 | YHC nurse and CP^a^ | Female | Municipal health service |
| 3 | 1 | 50 | YHC nurse and CP^a^ | Female | Municipal health service |
| 4 | 1 | 40 | YHC nurse and CP^a^ | Female | Municipal health service |
| 5 | 2 | 60 | Expert ‘Youth on a Healthier Weight’ (KnGG) | Female | Youth on a Healthier weight (JOGG) |
| 6 | 2 | 43 | Manager of the local integrated care approach | Female | Municipal health service |
| 7 | 2 | 50 | Expert ‘Youth on a Healthier Weight’ (KnGG) | Female | Youth on a Healthier weightw (JOGG) |
| 8 | 2 | 51 | Paediatrician | Male | Hospital |
| 9 | 2 | 63 | Professor of nutrition and health | Male | University |
| 10 | 2 | 35 | Manager of the local integrated care approach | Female | Municipal health service |
| 11 | 3 | 26 | Specialised YHC nurse^b^ | Female | Municipality |
| 12 | 3 | 59 | Specialised YHC nurse^b^ | Female | Municipality |
| 13 | 3 | 48 | YHC nurse^b^ | Female | Municipality |
| 14 | 3 | 47 | Director of Triple P Netherlands | Female | Municipal health service |
| 15 | 3 | 32 | Project leader of the local integrated care approach | Female | Municipal health service |
| 16 | 3 + 5 | 55 | Advisor | Female | Dutch Centre for Youth Health Care |
| 17 | 4 | 49 | Healthcare policy advisor | Female | Professional association |
| 18 | 4 | 49 | Paediatrician | Female | Hospital |
| 19 | 4 | 56 | Social worker | Female | Primary school |
| 20 | 4 | 30 | Dietician | Female | Dietician practice |
| 21 | 5 | 51 | YHC doctor | Female | Municipal health service |
| 22 | 5 | 35 | YHC doctor and researcher | Female | Municipal health service |
| 23 | 5 | 53 | Expert ‘Youth on a Healthier Weight’ (KnGG) | Male | Youth on a Healthier weight (JOGG) |
| 24 | 5 | 47 | Trainer and developer national education for CPs^a^ | Female | Municipal health service and self-employed |
| 25 | 5 | 41 | Advisor | Female | Dutch Centre for Youth Health Care |
| 26 | 5 | 30 | YHC doctor | Female | Netherlands Youth Institute |
| 27 | 5 | 28 | Advisor | Female | Netherlands Youth Institute |
| 28 | 5 | 48 | YHC nurse and CP^a^ | Female | Municipal health service |

^a^ CP: coordinating professional

^b^ YHC nurse: youth healthcare nurse

#### Distribution of positions

| Functions | Total | Percentage (%) |
| --- | --- | --- |
| YHC nurse^b^ | 6 | 17.1% |
| CP^a^ | 5 | 14.3% |
| Advisor | 4 | 11.4% |
| Expert‘Youth on a Healthier Weight’ (KnGG) | 3 | 8.6% |
| YHC doctor | 3 | 8.6% |
| Paediatrician | 2 | 5.7% |
| Project leader of the local integrated care approach | 2 | 5.7% |
| Manager of the local integrated care approach | 2 | 5.7% |
| Specialised YHC nurse^b^ | 2 | 5.7% |
| Professor of nutrition and health | 1 | 2.9% |
| Director of Triple P Nederland | 1 | 2.9% |
| Social worker | 1 | 2.9% |
| Dietician | 1 | 2.9% |
| Researcher | 1 | 2.9% |
| Trainer and developer of national education for CPs^a^ | 1 | 2.9% |
| Total | 35 | 100% |

^a^ CP= coordinating professional

^b^ YHC nurse= youth healthcare nurse

#### Distribution of organisations

| Organisation | Total | Percentage (%) |
| --- | --- | --- |
| Municipal health service | 12 | 41.4% |
| Municipality | 3 | 10.3% |
| Youth on a healthier weighthw (JOGG) | 3 | 10.3% |
| Hospital | 2 | 6.9% |
| Netherlands Youth Institute | 2 | 6.9% |
| Dutch Centre for Youth Healthcare | 2 | 6.9% |
| Dietician practice | 1 | 3.5% |
| University | 1 | 3.5% |
| Primary school | 1 | 3.5% |
| Professional association | 1 | 3.5% |
| Self-employed | 1 | 3.5% |
| Total | 29 | 100% |
